# Commentary: Analysis of risk factors for painful diabetic peripheral neuropathy and construction of a prediction model based on Lasso regression

**DOI:** 10.3389/fendo.2025.1519556

**Published:** 2025-03-31

**Authors:** Bingdi Wei, Yao Liu, Xiaorui Liu, Chaojun Wei

**Affiliations:** Gansu University of Chinese Medicine, Lanzhou, China

**Keywords:** painful diabetic peripheral neuropathy, type 2 diabetes mellitus, risk, LASSO regression, diabetic

## 1 Introduction

Diabetic peripheral neuropathy (DPN) is the most common complication of type 2 diabetes (T2DM), with a prevalence ranging from 30% to 90%, and 21% to 53.7% of patients experiencing painful DPN (PDPN), many of whom are undiagnosed (1, 2). As a form of neuropathic pain, PDPN severely affects patients’ quality of life, leading to sleep disturbances and depression. Its pathogenesis remains unclear but is thought to be related to glucose metabolism disorders, inflammation, and oxidative stress (3). While glycemic control plays a role in preventing DPN, its effectiveness for PDPN is limited, particularly in T2DM patients (4). Thus, early detection and prevention of PDPN are crucial (5). However, there is limited research on the prevalence and risk factors of PDPN in China, with no available data from Hunan Province. Yu et al. evaluated the prevalence and risk factors of PDPN among T2DM patients in Hunan Province and developed a predictive model, providing valuable new insights for PDPN risk prediction, although some limitations remain (6).

## 2 Commentary and discussion

This study focuses on the occurrence and risk factors of painful diabetic peripheral neuropathy (PDPN) in patients with type 2 diabetes mellitus (T2DM) and constructs a prediction model for PDPN based on the Lasso regression method. PDPN is a common and impactful complication of diabetes, often accompanied by significant chronic pain that severely affects patients’ quality of life. Therefore, since the risk factors of PDPN are not yet fully understood, identifying and quantifying these risk factors is of great clinical significance for early intervention and individualized management of patients.

This article mainly discusses the innovative methods and limitations of the study, particularly the advantages and disadvantages in terms of variable selection, sample representativeness, data quality, and methodology. Although the study demonstrated methodological innovation, its limitations in variable comprehensiveness, sample applicability, and external validity of the model may restrict its broader application. Therefore, this article proposes suggestions for further research improvements based on in-depth analysis.

To select variables, this study employed Lasso regression, a method that is relatively uncommon in traditional PDPN research. Lasso regression reduces the multicollinearity problem and eliminates redundant variables through penalty coefficients, allowing the model to maintain good predictive power even in high-dimensional data. Lasso regression is a type of linear regression method that works by adding a penalty term to the loss function, which is proportional to the absolute value of the regression coefficients. This regularization technique helps to shrink some of the coefficients to zero, effectively performing both variable selection and regularization, thereby improving model interpretability and preventing overfitting (7).

The key advantage of Lasso regression is its ability to automatically select important features by shrinking the coefficients of irrelevant ones to zero, simplifying the model and preventing overfitting. This makes it particularly suitable for high-dimensional data with fewer samples. Compared to linear regression, Lasso offers better prevention of overfitting; compared to Ridge regression, it performs feature selection for a more streamlined model; and compared to Support Vector Regression, Lasso is more computationally efficient and easier to interpret (7, 8).

In addition, This study is rigorously designed, dividing data from 4908 patients into training and validation sets, and employs ROC curves, calibration curves, and decision curve analysis (DCA) to evaluate model performance from multiple perspectives, ensuring the stability and reliability of the prediction model. However, when using DCA to assess the clinical utility of the model, it is important to note that DCA results may be influenced by the choice of threshold probabilities, and DCA alone may not fully capture the incremental value of the model compared to existing clinical practices (9). To provide a more comprehensive evaluation, future studies are recommended to incorporate additional metrics, such as net reclassification improvement (NRI) and integrated discrimination improvement (IDI), to quantify the model’s improvements in patient risk reclassification and risk discrimination capabilities, thereby more thoroughly validating the clinical utility of the model.

However, there are still some limitations in the study. First, the study is based on patient data from a single center in Hunan Province, which has geographical and population-specific limitations and may not broadly represent the PDPN risk situation of populations from different regions and ethnicities. Differences in lifestyle habits and genetic backgrounds across regions may lead to significant differences in PDPN incidence and risk factors, thus limiting the external validity of the model.

Secondly, as a retrospective study, this research can only reveal correlations between variables and cannot determine causality. For example, although smoking was found to be significantly associated with PDPN, this does not directly indicate that smoking is a causative factor for PDPN (10). The lack of causal relationships limits the study’s applicability in clinical interventions and may lead to imperfections in the design of intervention strategies. The original study excluded patients with a history of mental illness, cancer, cervical or lumbar spine lesions (e.g., nerve compression, spinal stenosis), cerebral infarction, lower limb vascular occlusion, alcoholism, connective tissue disease, as well as pregnant or lactating patients, without providing specific rationale for these exclusions. Potential reasons may include data limitations, such as uncollected or incompletely recorded variables (e.g., behavioral or psychological factors), or the study’s focus on specific risk factors (e.g., physiological indicators) while overlooking others. These missing variables could impact the model’s predictive ability, and future studies should consider incorporating them, where data permits, to further optimize the model’s accuracy and clinical utility.

Thirdly, although the study considered smoking as a lifestyle variable, it did not include other factors such as alcohol consumption, exercise habits, and mental health (11). Specifically, PDPN patients often experience significant psychological and emotional burdens, such as anxiety and depression, which not only affect patients’ pain perception but may also profoundly influence the occurrence and progression of PDPN (12). Therefore, the lack of analysis of mental health factors such as anxiety and depression may underestimate their impact on PDPN, leading to limitations in the comprehensiveness and predictive accuracy of the model in assessing PDPN risk. Including these variables in the analysis could improve the model’s predictive accuracy and clinical utility.

Additionally, the occurrence of PDPN is not only related to health status but may also be influenced by specific treatment methods, such as different hypoglycemic drugs and blood glucose control strategies (13). However, the study did not include information on patients’ treatment regimens, which may limit the model’s applicability to individualized management. Including treatment regimens as model variables could reveal more about the influence of therapeutic factors on PDPN, thereby enhancing the model’s reference value in actual treatment processes.

The use of retrospective data in this study may result in incomplete or missing information, potentially introducing selection bias, recall bias, and issues related to missing data. Selection bias may arise if the included subjects are not representative of the broader population, potentially skewing the findings. Recall bias could occur if patients’ recollections of past medical history or exposures are inaccurate, affecting the reliability of the data. Additionally, missing data, if not handled properly, can introduce systematic errors, leading to biased conclusions. The study did not provide details on the strategies for data cleaning and bias control, which could affect the robustness of the results. Proper techniques, such as multiple imputation for missing data, and more rigorous methods for minimizing selection and recall bias, would improve the scientific rigor and credibility of the study, ensuring more accurate and reliable conclusions.

Genetic factors related to PDPN were also not considered in this study. Recent research indicates that genetic variations have a significant impact on diabetes complications, particularly the susceptibility to PDPN (14). Including genetic data could help identify genetically susceptible individuals, thereby providing a personalized risk assessment plan for patients.

Socioeconomic status (SES) is also an important factor influencing health behaviors and access to medical care, potentially affecting management outcomes for PDPN. However, the lack of SES factors, such as income and education level, may limit the model’s ability to identify high-risk groups (15). Including SES could optimize the model’s applicability across different social groups, particularly in regions with significant socioeconomic disparities.

Furthermore, the study did not model symptom severity in PDPN patients, even though it may significantly impact management needs and intervention outcomes (16). Future studies could consider including PDPN grading as a predictive variable to develop more individualized treatment and management plans based on different severity levels, thereby improving the model’s clinical applicability.

Introducing a novel approach to predicting PDPN risk, this study utilizes Lasso regression, making a significant contribution to diabetic complications research. The findings offer crucial theoretical support for clinicians in identifying high-risk PDPN patients, facilitating personalized management and intervention strategies. The developed model serves as an effective clinical tool, helping physicians implement early interventions for high-risk individuals, ultimately improving their quality of life. Moreover, the study identifies several potential risk factors for PDPN, providing a solid foundation for the comprehensive management of diabetic patients.

To overcome the limitations of the current study, future research could collect data from different regions and ethnic backgrounds to improve the model’s generalizability and external validity. To better validate causal relationships, future studies are recommended to conduct prospective cohort studies that dynamically track the progression of patients’ conditions, thereby more accurately revealing the causal effects of various risk factors on PDPN. Furthermore, it is suggested to include psychological factors as independent variables in the PDPN prediction model to reveal their comprehensive impact on PDPN and enhance the model’s predictive accuracy and applicability. Adding more lifestyle, treatment, genetic, and SES variables to the model can also enhance its comprehensiveness and predictive capability. Future studies could also consider incorporating PDPN severity as a variable to more precisely guide clinical interventions and develop individualized treatment and management plans.

In conclusion, this study provides an innovative model and valuable reference for PDPN risk prediction, but there are still deficiencies in sample representativeness, causal verification, variable comprehensiveness, and external applicability of the model. Future research can improve the model by collecting multi-center data, including multidimensional variables, and adopting prospective designs to enhance its clinical application value. These improvements will not only support individualized management for diabetic patients but also promote further development of PDPN prediction methods, driving the advancement of precision medicine in the field of diabetes complications.
